# The Effects of Inorganic Salts with Different Anions on the Structure and Properties of Starch/Poly (Butylene Succinate) Blends Plasticized with Ionic Liquid

**DOI:** 10.3390/polym11122004

**Published:** 2019-12-03

**Authors:** Zhixin Zhao, Bei Lei, Wenhao Du, Xi Zhang

**Affiliations:** 1 State Key Laboratory of Polymer Materials Engineering, Sichuan University, Chengdu 610065, China; zzx0830@126.com (Z.Z.); LeiB2230@163.com (B.L.); dwhde740956779@163.com (W.D.); 2 Polymer Research Institute, Sichuan University, Chengdu 610065, China

**Keywords:** poly(butylene succinate), starch, inorganic salts, ionic liquid, anion, modification

## Abstract

1-butyl-3-methylimidazole chloride ((BMIM) Cl) modified starch/poly (butylene succinate) (PBS) blends with different anions of inorganic salts were prepared by HAAKE mixer. The compatibility and thermal behaviors including crystallinity, crystallization and melting temperature, thermal stability and mechanical properties were systematically investigated by Fourier transform infrared spectroscopy (FT-IR), scanning electron microscopy (SEM), thermogravimetric analysis (TGA), differential scanning calorimetry (DSC), and X-ray diffraction (XRD). The results show that inorganic salts can produce a strong interaction with starch/PBS blends, which can improve the mechanical properties of starch/PBS blends, enhance the mechanical strength and elongation at break of starch/PBS blends, meanwhile, the thermal stability of starch/PBS blends decreased. The SEM images reveal that the compatibility of starch/PBS become better with the increase of inorganic salts. The melting and crystallization absorption peaks in the DSC curves show that the melting enthalpy (Δ*H*_m_), crystallinity (*X*_c_), and crystallization temperature (*T*_c_) of the blends decrease and the cold crystallization temperature (*T*_cc_) increase when inorganic salts is added. Moreover, inorganic salts with smaller anionic radius have much better effects on the starch/PBS blends plasticized with ((BMIM)Cl).

## 1. Introduction

As the continuous consumption of petroleum resources of raw materials for the preparation of polymer materials, and the increasing emphasis on environmental pollution caused by the difficult degradation of waste polymer materials, the development of eco-friendly polymer materials based on natural and non-petroleum resources has been greatly concerned by the governments and scientific research institutions all over the world [1,2,3]. Starch is a non-polluting, inexpensive natural degradable polymer material. PBS is one of the typical fully biodegradable materials which can be obtained by fermentation of biological resources. Polylactic acid (PLA) [4,5,6], polycaprolactone (PCL) [7,8,9], and poly(3-hydroxybutyrate) (PHB) [10,11,12] are typical biodegradable aliphatic polyesters, PBS stands out for the excellent performance and good industrial applications, such as eco-friendly nature, excellent properties in terms of biodegradability, processability, thermal properties and chemical resistance, and becomes one of the fastest growing varieties [13,14,15].

Starch is a highly abundant, non-toxic, and renewable biopolymer, which is produced from assimilating plants. It is extensively utilized in various fields, such as food, paper, wood, and pharmaceutical industry [16,17,18,19], and starch gradually becomes the main green chemical raw materials [20,21,22]. Therefore, the starch and PBS are mixed and the composite is expected to be eco-friendly with low cost and good performance. However, PBS exhibits hydrophobicity for the repeated aliphatic units on its main chain while starch exhibits strong hydrophilicity due to its crystalline structure and a large number of hydrogen bonds of intra-molecular and inter-molecular, which lead to the bad compatibility between PBS and starch [23,24,25,26,27]. Meanwhile, the interface bonding strength between the untreated starch particles and PBS interface is weak and the heat resistance, water resistance, and mechanical properties are terrible, hence, traditional simply blending can impossibility produce the ideal composite materials [1,28,29]. 1-butyl-3-methylimidazolium chloride ((BMIM)Cl) is a kind of ionic liquid, which can plastify and modify starch/PBS blends. By adding ((BMIM)Cl), the compatibility of starch/PBS and the brittleness of the composites greatly improve, however, the strength of the starch/PBS blends immensely reduced after plasticizing process.

In our work, we use extra magnesium sulphate (MgSO_4_) and magnesium chloride (MgCl_2_) inorganic salts cooperatively with the ((BMIM)Cl) to modify the starch/PBS blends, in addition, the different effects of MgSO_4_ and MgCl_2_ on the structure and properties of starch/PBS were studied. There are abundant oxygen atoms in starch and PBS, the ions of inorganic salt can interact with oxygen atoms and the interaction can change the aggregation structure of starch/PBS blends, the bonding strength of “macromolecule-modifying agent-macromolecule” in starch and starch/PBS blends are regulated which can inhibit the strength reduction from the plastication of ((BMIM)Cl) and improve the compatibility of starch/PBS blends. This method provides a new idea for the modification of starch/PBS blends.

## 2. Materials and Methods 

### 2.1. Materials

Poly(butylene succinate) was kindly provided from Anqing Hexing Chemical Co., Ltd. (Anqing, China), which was injected in the molding grade. Corn starch was purchased from Xiadian corn development Industrial Co., Ltd. (Xi’an, China), which was in an edible level. Ionic liquid ((BMIM)Cl) (purity 97%) was purchased from Aladdin Biochemical Technology Co., Ltd. (Shanghai, China), which was used without any further purification. Magnesium chloride (MgCl_2_) and Magnesium sulphate (MgSO_4_) were purchased from Kelong Chemical Co. (Chengdu, China), which was in analytical purity.

### 2.2. Sample Preparation

The samples were prepared by the melt blending method. The starch and PBS were dried at 105 °C for 5 h under vacuum oven (*p* < 0.3 MPa). Starch and plasticizer (Ionic liquid and inorganic salts) were pre-mixed at room temperature for 2 h, and placed in a vacuum oven at 105 °C for 72 h before blending. The mixture was further plasticized in HAAKE Rheomix OS Torque Rheometer (Thermo Electron GmbH, Karlsruhe, Germany) at 125 °C with a rotor speed of 70 rpm for 15 min.

Starch/PBS blends were then prepared by melting thermoplastic starch and PBS in a HAAKE Mixer at 125 °C with a rotor speed of 70 rpm for 15 min. The compositions of samples were listed in Table 1 and Table 2 (according to the content of different inorganic salts, a series of samples were called as TSP, TSPMS1, TSPMS2, TSPMS3, TSPMS4, TSPM1, TSPM2, TSPM3, TSPM4 for short.). Finally, the blends were processed with an injection molding machine to obtain specimens for mechanical and other measurements. The barrel and mold temperatures during injection molding were 125 and 25 °C, respectively. The injection and packing pressure were both 400 bar. Dumb-bell specimens (width 4 mm, thickness 2 mm, length of parallel part 30 mm, and total length 75 mm) were prepared for the tensile tests.

### 2.3. Characterizations

#### 2.3.1. Fourier Transform Infrared (FT-IR) Spectroscopy

The samples were dried in a vacuum oven at 105 °C for 5 h to remove water and then crushed into powder with a pulverizer. The infrared spectra were measured using a Fourier transform infrared spectrophotometer (Nicolet 560, Welltech Scientific Inc, Chantilly, VA, USA), at spectral resolution of 4 cm^−1^ and a scanning range from 400 to 4000 cm^−1^.

#### 2.3.2. Scanning Electron Microscopy (SEM)

The morphology of the samples was examined with a scanning electron microscope JSM-5900LV (JEOL, Tokyo, Japan). The surfaces were coated with platinum prior to examination. The accelerating voltage was 10 kV.

#### 2.3.3. Thermal Gravity Analysis (TGA)

TGA was performed by the TA2950 TGA thermal analysis instrument (Du Pont China Holding Co., Ltd, Beijing, China). The samples for testing were heated from room temperature to 600 °C at a heating rate of 20 °C min^−1^.

#### 2.3.4. Differential Scanning Calorimetry (DSC)

The DSC measurements were performed by a differential scanning calorimeter (DSC8500, PerkinElmer Inc, Billerica, MA, USA). Samples (3–5 mg) were placed in sealed aluminum crucibles under a flow of nitrogen. Each sample was first heated from 30 to 140 °C at a rate of 50 °C min^−1^, kept at 140 °C for 3 min to eliminate thermal history, cooled to 30 °C at a cooling rate of 10 °C min^−1^, kept at 30 °C for 2 min, and then reheated at a rate of 10 °C min^−1^ to 140 °C.

#### 2.3.5. X-ray Diffraction (XRD) Measurements

The XRD test was carried out by using an X’Pert Pro MPD diffractometer (Philips Analytical Company, Eindhoven, The Netherlands), with nickel filter CuKa as the radiation source. The acceleration voltage was 50 kV and the current was 35 mA. The samples were continuously scanned over the 2θ range from 5° to 50°.

#### 2.3.6. Mechanical Properties Test

The mechanical properties (tensile strength (TS), elongation at break (%Eb) and Young’s modulus) of the samples were determined using a tensile testing equipment (Instron 5567, Instron Inc, Boston, MA, USA). The crosshead speed was 20 mm min^−1^. The initial gauge length of the specimen was 20 mm. The width of each tensile sample was 4 mm. All samples were balanced in 30% relative humidity (RH) at 25 °C for 48 h before the test. Five specimens were used for each group, and the average values plus the standard deviation were determined. *t*-Test was carried out to determine the statistically significant difference (*p* < 0.05) among the mechanical properties using STATA (STATA 11.2 for windows).

## 3. Results and Discussion

### 3.1. FT-IR Analysis

The interaction between anion and cation of inorganic salts and the hydroxy(-OH) of starch and PBS is the main interaction between inorganic salts and TSP composite. The movement of the stretching vibration peak of –OH can reflect the intensity of the electron interaction. Therefore, FT-IR was used to characterize the TSP before and after the modification by inorganic salts. The TSP sample without any addition of inorganic salt shows a peak at 3411 cm^−1^ which corresponds to the stretching vibration absorption peak of starch and PBS molecular chains [30], a peak at 1084 cm^−1^ corresponds to the stretching vibration peak of C–O in the C–O–H group of the starch molecules, and a peak at 1028 cm^−1^ corresponds to the stretching vibration peak of C–O in C–O–C of the starch molecular chain, and a peak at 1723 cm^−1^ corresponds to the stretching vibration absorption peak of C–O in the ester group of the PBS molecules [31].

**Figure 1:**
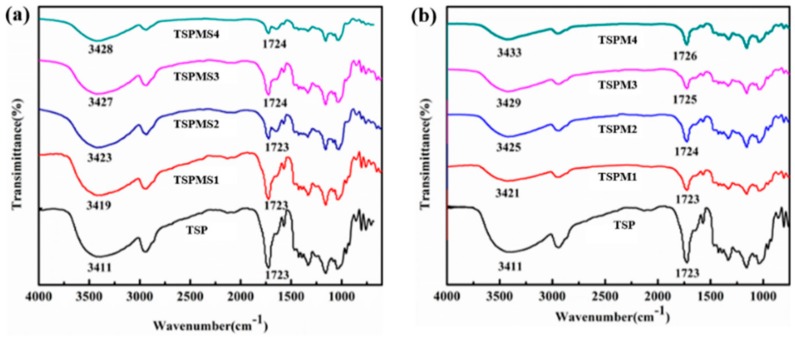
The FTIR spectra of TSP blends modified with MgSO_4_ (**a**) and MgCl_2_ (**b**).

**Figure 2:**
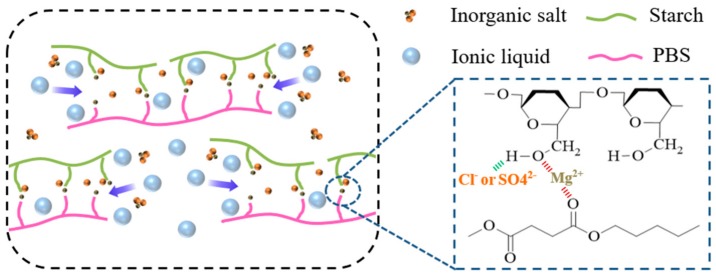
The mechanism of TSP blends modified with different inorganic salts.

Figure 1 shows the IR spectra of the TPS added with MgSO_4_ and MgCl_2_, and no extra absorption peaks of new chemical groups, which indicates that both MgSO_4_ and MgCl_2_ did not react with the matrix. As shown in Figure 1a,b, the –OH and C–O peaks of TSP modified by MgSO_4_ and MgCl_2_ shift to the high wavenumber, which indicates that the inorganic salt has strong interaction with starch and PBS molecular chains. Additionally, Figure 2 reveals the mechanism that electron-rich Cl^−^ or SO_4_^2−^ formed an electronic interaction with oxygen atoms in the hydroxyl group of starch molecules and in the ester group of PBS molecules, while the electron-deficient Mg^2+^ can interact with the hydrogen atoms in the hydroxyl group of both starch and PBS molecules with the separation role of ionic liquid. Therefore, inorganic salts can weaken the hydrogen bonds and lead to the change of aggregation structure and properties of the TSP. Furthermore, the comparison of TSPMS and TSPM shows that the effect of MgCl_2_ on the blue shift of hydroxyl peak in starch and PBS was more obvious than that of MgSO_4_, as the addition of both inorganic salts were the same. Meanwhile, the comparison between TSPMS and TSPM shows that the MgCl_2_ had a greater effect on the blue shift of –OH than MgSO_4_, which indicates that Cl^−1^ has a more pronounced effect on the –OH peak of TSP than SO_4_^2−^. This is probably because the ion radius of SO_4_^2−^ is larger than that of Cl^−^, thus the interaction between SO_4_^2−^ and hydroxyl groups on TSP is weaker than that of Cl^−^, which eventually leads to the movement of stretching vibration absorption peak of –OH. The smaller the ionic radius of the ion is, the stronger the interaction between ions and –OH, the more obvious the infrared blue shift of hydroxyl. 

In summary, inorganic salts can further affect the absorption peaks of hydroxyl and ester groups in TSP. The ionic radius is the main factor which can impact the hydroxyl peaks in TSP. The smaller the ionic radius of inorganic ions, the stronger the ability of ions to enter the molecular chains between starch and PBS, and the more obvious the influence on the movement of hydroxyl and ester groups peaks.

### 3.2. Scanning Electron Microscopy

As the mechanical properties are strongly dependent on the microscopic morphologies, the performance and properties of blending materials not only depend on the properties of the individual components but also their interfacial compatibility, so increasing the compatibility between the components is an important way to improve the performance of the materials.

**Figure 3:**
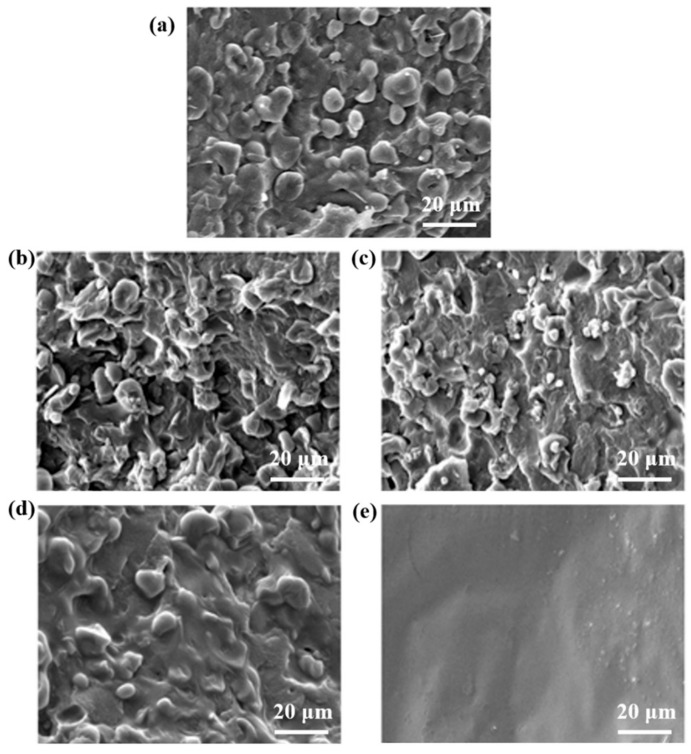
The SEM micrographs of TSP (**a**), TSPMS1 (**b**), TSPMS4 (**c**), TSPM1 (**d**), TSPM4 (**e**).

In order to investigate the effect of inorganic salts on the compatibility of TSP blends, Figure 3 shows the SEM images of the cross section of the TSP blends with different content of inorganic salts. As shown in Figure 3a, the interface in the pure TPS sample is obvious, the starch particles inhomogeneously disperse in the continuous phase of the PBS matrix then forms the sea-island structure, the adhesion between the two-phase is very weak and there are some starch granules fall off from the section, and the grooves formed. This is due to the incomplete separation effect of the plastication of ionic liquid during the dissolution process, some starch granules retain with a low compatibility with PBS, resulting in an obvious phase separation structure. By comparing Figure 3b–e, it can be seen that samples gradually show no obvious interface between the two phases with the increase of inorganic salts, the amount of starch granules fall off from the matrix reduces and the adhesion of the two phases enhances. Furthermore, Figure 3 shows that there are still some residual starch granules and cracks in the fractured surfaces of the TSPMS4 blends, while the TSPM4 blends exhibited homogeneous and continuous morphology without visible starch granules and aggregation.

In general, the compatibility of starch/PBS blends plasticized with IL is further improved by the addition of inorganic salts. As the inorganic salts interfere in the formation of hydrogen bonds within the starch and PBS matrix and disturb the crystallization of the starch and PBS. Meanwhile, the electron-rich Cl^−^ with small ionic radius is stronger than that of SO_4_^2−^, hence the electronic interaction with hydroxyl groups of starch molecular chains and ester groups of PBS molecular chains is strong.

### 3.3. Thermal Stability Analysis

**Figure 4:**
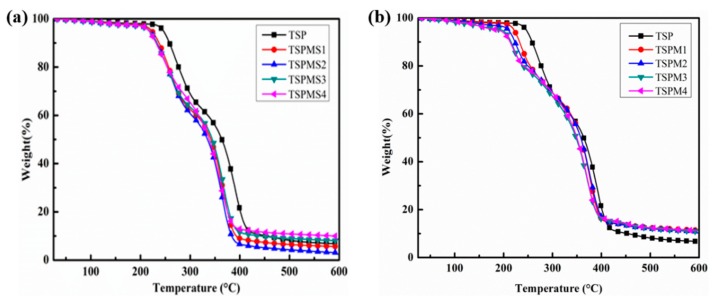
The TGA curves of starch/PBS blends modified with MgSO_4_ (**a**) and MgCl_2_ (**b**).

The effect of inorganic salts on the thermal stability of starch/PBS composite system was studied by thermogravimetric analysis, as Figure 4a,b shown the TGA curves of MgSO_4_ and MgCl_2_ modified starch/PBS composite samples, respectively. The corresponding initial thermal decomposition temperatures (*T*_i_) are listed in Table 3. All the results reveal that the *T*_i_ of each sample shows a decreasing tendency along with the increase of inorganic salt, moreover, the MgCl_2_ modified starch/PBS composite sample shows a much lower T_i_ than the MgSO_4_ modified sample. On the other hand, the *T*_i_ of the composite sample decreases to 215 °C as the content of MgSO_4_ is 4 wt%, while the *T*_i_ of the MgCl_2_ modified sample is 203 °C. This is due to the MgSO_4_ and MgCl_2_ weak the hydrogen bonds between the starch and PBS molecules and damage the perfect degree of crystallization of starch/PBS system, thus weak the thermal stability. In addition, the electronic interaction between MgSO_4_ and starch/PBS system is weaker than the MgCl_2_ modified sample, hence MgSO_4_ shows less impact on the thermal stability of the blend system.

In general, as the inorganic salts interfere the formation of hydrogen bonds within the starch and PBS matrix and disturb the crystallization of the starch and PBS. The *T*_i_ of each sample shows a decreasing tendency. Moreover, the electron-rich Cl^−^ with small ionic radius is stronger than that of SO_4_^2−^, hence the electronic interaction with hydroxyl groups of starch molecular chains and ester groups of PBS molecular chains become stronger and the MgCl_2_ modified starch/PBS composite sample shows a much lower thermal stability.

### 3.4. Thermal Properties

In order to further study the interaction between MgSO_4_ or MgCl_2_ and starch /PBS, the inorganic salt modified starch /PBS blend samples were tested by DSC. Figure 4 shows the DSC curves of modified starch/PBS composite with different ratio of MgSO_4_ and MgCl_2_, respectively. The melting enthalpy corresponding *Δ*(*H_m_*), crystallinity (*X_c_*), the crystallization temperature (T_c_), and the cold crystallization temperature (T_cc_) are listed in Table 4. *X_c_* is calculated as Equation (1):

(1) $$Xc\%=100\%\times \frac{\Delta Hm}{\Delta H_{m}^{0}\times wt}$$

where *ΔH_m_* represents the melting enthalpy of the corresponding sample, *ΔH^0^_m_* is the melting enthalpy of one hundred percent crystallization, and wt is the content of PBS.

**Figure 5:**
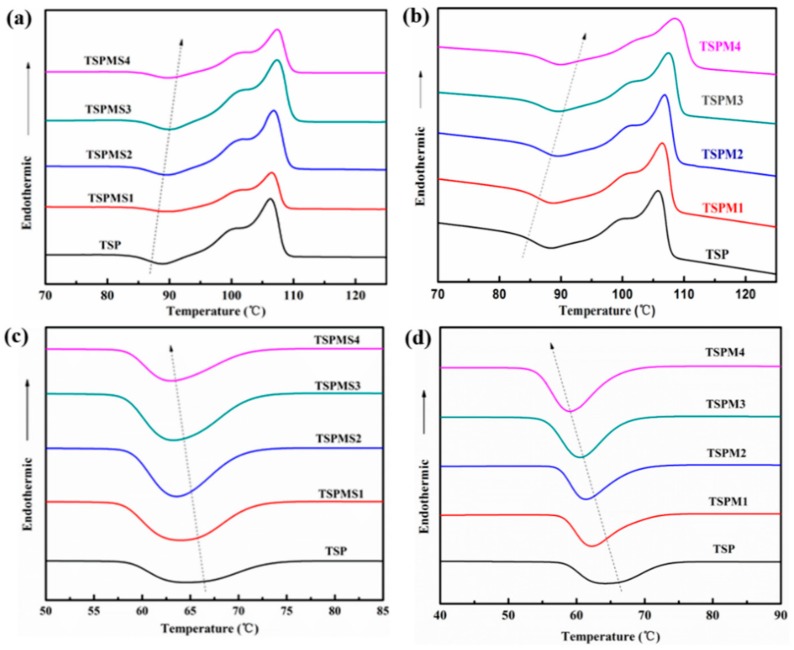
The DSC melting curves of TSP blends modified with MgSO_4_ (**a**) or MgCl_2_ (**b**); the DSC crystallization curves of TSP blends modified with MgSO_4_ (**c**) or MgCl_2_ (**d**).

Figure 5 and Table 4 reveal that the *ΔH_m_*, *X_c_* and Tc of starch/PBS composites decrease while the *T*_cc_ increase as the content of MgSO_4_ and MgCl_2_ increased. This is due to the strong interaction between MgSO_4_ and the molecular chain of starch and PBS, as the Mg^2+^ tend to form the electronic interaction with the oxygen atoms of hydroxy in starch chains and oxygen atoms of ester in PBS chains, which make the force of molecular inter-atomic strong and restrict the movement of chains, hence raise the energy of the rearrangement and the temperature of cold crystallization. The addition of MgSO_4_ enhances the interaction between PBS and starch molecules, so relatively hindered the orderly arrangement in PBS chains and disturb the perfect degree of crystallization. Hence, the Δ*H*_m_ and corresponding *X*_c_ decrease, meanwhile, the chain segment is difficult to pile up and requires great driving force, and the crystallization temperature decreases.

The effect of MgCl_2_ on thermal properties of starch/PBS composite is similar to the MgSO_4_, as shown in Figure 5b,d and Table 4, indicating that MgSO_4_ and MgCl_2_ have the similar rules. The thermal performance parameters of the MgSO_4_ blending system changes more slightly than that of MgCl_2_ as the adding amount was 4 wt%, by contrasting Figure 5a,c and Table 4. As the *X*_c_ and *T*_c_ of MgSO_4_ blending system decrease to 39.5% and 63.2 °C, respectively, and the *T*_cc_ raises to 89.8 °C, while the *X*_c_, *T*_c_, and *T*_cc_ of the corresponding MgCl_2_ system change to 37.8%, 59.2 and 91.7 °C, respectively (this conclusion is only suggested by our test results.) This is mainly because the participation efficiency of ions in MgSO_4_ is low, as only cation participates in the formation of strong electron interaction, while the electron interaction between SO_4_^2−^ and starch /PBS is very weak for the volume of SO_4_^2−^ is huge which restricts the electron interaction. Additionally, MgCl_2_ shows strong electron interaction as both cation and anion can easily participate in the formation of electron interaction. 

### 3.5. Crystalline Properties

The previous studies have proved that inorganic salts could play the role of plasticizer by disturbing the crystallization of starch/PBS blends [32]. Both the anions and cations in inorganic salts could form the strong coordination effect with –OH. These interactions would interrupt the intermolecular and intramolecular hydrogen bonding of starch and PBS to disturb the growth of crystal. The effect of inorganic salts on the crystalline property of starch/PBS blends was studied by XRD.

**Figure 6:**
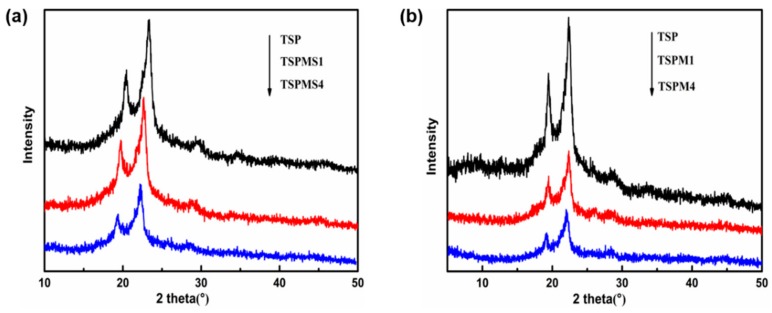
The XRD diffractograms of TSP blends modified with MgSO_4_ (**a**) or MgCl_2_ (**b**).

Figure 6a,b show the X-ray diffraction spectra of the TSP blends and TSP blends modified with different inorganic salts. The previous studies have proved that PBS has three diffraction peaks when 2θ angles are 19.5, 21.5, and 22.5°, corresponding to (020), (021), and (110) crystal planes [33,34,35], respectively. It could be seen from Figure 6 that the diffraction peak positions of the TSP blends do not move or change as the increase of the MgSO_4_ or MgCl_2_. There is no new crystal diffraction peaks that appear, indicating that the addition of inorganic salts does not alter the crystalline form of PBS.

Moreover, the crystallization peak intensity of the TSP blends obviously reduced with the increase of inorganic salts. This phenomenon indicated that MgSO_4_ or MgCl_2_ had a damaging effect on the crystals of TSP blends and form strong interaction with TSP blends molecules, the interaction could interrupt the hydrogen bonding in the crystalline structure of TSP blends. In addition, the intensity of the TSPM crystal peak was weaker than that of the TSPMS crystal peak. This is because the ionic radius of Cl^−1^ is smaller than that of SO_4_^2−^, and Cl^−1^ can more easily enter into the molecular chains of the starch and PBS and break the crystallization. This is also consistent with the results of DSC. It could also be noticed that the diffraction peaks of MgSO_4_ and MgCl_2_ did not appear in Figure 6a,b, respectively. This also suggests that MgSO_4_ and MgCl_2_ were completely dissolved in TSP blends and no MgSO_4_ and MgCl_2_ crystals or congregates existed.

### 3.6. Mechanical Properties

**Figure 7:**
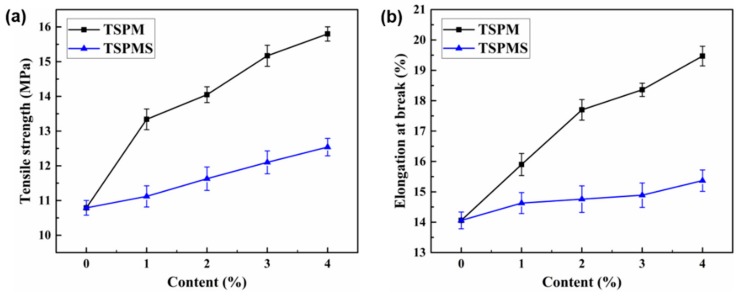
(**a**) The tensile strength of TSP blends modified with MgSO_4_ or MgCl_2_; (**b**) the elongation at break of TSP blends modified with MgSO_4_ or MgCl_2_.

The influence of inorganic salts on the mechanical properties of TSP blends was evaluated by tensile testing. As the starch contains a large number of intermolecular and intramolecular hydrogen bonds, it is easy to form microcrystals, which makes the starch and starch-based materials show strong brittleness. Figure 7 shows the tensile strength and elongation at break of TSP blends before and after being modified with various content of MgSO_4_ and MgCl_2_. Table 5 shows the mechanical properties (tensile strength, elongation at break and Young’s modulus) of TSP, TSPMS, and TSPM blends. After the addition of the inorganic salts, the tensile strength and the elongation at break of the TSP blends increased obviously. This is due to the fact that both anions and cations of the inorganic salts have strong interaction with starch and PBS molecular chains hence improve the mechanical properties of the blends. Moreover, the electron-rich Cl^−^ with smaller ionic radius has higher anionic utilization efficiency than that of SO_4_^2−^, which may have stronger interaction with starch/PBS molecular chains. These result in the tensile strength and the elongation at break of the TSPM blends increasing more than that of TSPMS blends, this is consistent with the previous results in this article. Without adding inorganic salts, the tensile strength of TSP was 10.8 MPa, the elongation at break was 14.1%, which showed obvious brittleness. After adding 4 wt% MgCl_2_, the tensile strength increased to 15.8 MPa and the elongation at break increased to 19.5%, which increased 46% and 38%, respectively than that without adding MgCl_2_. Meanwhile, the tensile strength increased to12.5 MPa and the elongation at break increased to 15.4% with the addition of 4 wt% MgSO_4_ (this conclusion is only suggested by our test results.) The elongation at break of all the TSPMS and TSPM blends maintained at a high level, which is rarely observed in previous researches. This is because the addition of inorganic salts weakens the original hydrogen bonds in starch and PBS molecules. Electron-rich anions can interact strongly with oxygen atoms in starch/PBS blends to enhance the compatibility of starch and PBS phases, thus improving the mechanical strength and flexibility of the composites. It was worth noting that the tensile strength and elongation at break of the TSP blends modified by MgCl_2_ were both higher than that of MgSO_4_. This is because the Cl^−^ in MgCl_2_ has more smaller ion radius and form stronger electronic interaction, the performance is improved more obviously. From all the above, we can conclude that inorganic salts can compensate the loss of mechanical strength caused by the plasticizers and provide a new method to control the properties of starch/PBS blends composites, especially, when using inorganic salts with the same cations to modify the blends, the anionic parts of inorganic salts with small volume can be preferentially selected.

## 4. Conclusions

In summary, the ((BMIM)Cl) modified starch/PBS blends were further modified by MgSO_4_ and MgCl_2_, in which the electron-rich anions SO_4_^2−^ and Cl^−^ form electronic interaction with the oxygen atoms in the hydroxyl groups of starch and the ester group of PBS and destroy the original hydrogen bonds in the molecules. Compared to starch/PBS blends modified only with ((BMIM)Cl), the samples with the addition of inorganic salts can further break the ordered arrangement of the molecular chains in the crystal regions of both starch and PBS, resulting in a decrease in crystallinity and making the compatibility of starch and PBS significantly improve. With the increase of the inorganic salts, the melting enthalpy (Δ*H*_m_), crystallization temperature (*T*_c_), and the crystallinity (*X*_c_) decrease, while the cold crystallization temperature (*T*_cc_), the tensile strength, and elongation at break of starch/PBS blends increase. This is due to the electron interaction between inorganic salts and starch/PBS blends. This method of adding inorganic salts can compensate the loss of mechanical strength caused by ((BMIM)Cl) modification, and MgCl_2_ has a more significant effect on starch/PBS for the small anionic radius.

## Figures and Tables

**Table 1:** Mixing formulation of starch/PBS samples modified with MgSO_4._

Samples	Starch/g	PBS/g	(BMIM)Cl/g	MgSO_4_/g
TSP	16	24	4	0
TSPMS1	16	24	4	0.4
TSPMS2	16	24	4	0.8
TSPMS3	16	24	4	1.2
TSPMS4	16	24	4	1.6

**Table 2:** Mixing formulation of starch/PBS samples modified with MgCl_2._

Samples	Starch/g	PBS/g	(BMIM)Cl/g	MgCl_2_/g
TSP	16	24	4	0
TSPM1	16	24	4	0.4
TSPM2	16	24	4	0.8
TSPM3	16	24	4	1.2
TSPM4	16	24	4	1.6

**Table 3:** The thermal stability parameters of TSP blends modified with MgSO_4_ and MgCl_2_.

Sample	*T*_i_ (°C)	Residue at 600 °C (%)
TSP	243	6.8
TSPMS1	224	8.1
TSPMS2	221	5.6
TSPMS3	219	3.1
TSPMS4	215	9.9
TSPM1	218	11.4
TSPM2	214	10.7
TSPM3	204	12.5
TSPM4	203	12.6

**Table 4:** The DSC data of TSP blends modified with MgSO_4_ or MgCl_2_.

Sample	Δ*H_m_* (J/g)	*X_c_* (%)	*T_c_* (°C)	*T_cc_* (°C)
TSP	28.6	47.6	64.2	88.3
TSPMS1	26.9	45.1	64.0	88.9
TSPMS2	25.3	42.8	63.6	89.3
TSPMS3	24.4	41.6	63.4	89.7
TSPMS4	23.0	39.5	63.2	89.8
TSPM1	25.1	42.1	62.2	89.0
TSPM2	24.4	41.3	61.4	89.7
TSPM3	23.0	39.3	60.4	90.1
TSPM4	22.0	37.8	59.2	91.7

**Table 5:** The mechanical properties of TSP blends modified with MgSO_4_ or MgCl_2_.

Sample	Tensile Strength (MPa)	Elongation at Break (%)	Young’s Modulus (MPa)
TSP	10.8 ± 0.21	14.1 ± 0.27	687 ± 5.36
TSPMS1	11.1 ± 0.30	14.6 ± 0.34	768 ± 6.68
TSPMS2	11.6 ± 0.33	14.8 ± 0.43	839 ± 8.36
TSPMS3	12.1 ± 0.32	14.9 ± 0.40	951 ± 7.52
TSPMS4	12.5 ± 0.25	15.4 ± 0.35	1052 ± 6.57
TSPM1	13.3 ± 0.29	15.9 ± 0.36	827 ± 5.54
TSPM2	14.1 ± 0.22	17.7 ± 0.33	943 ± 9.34
TSPM3	15.2 ± 0.30	18.4 ± 0.21	1001 ± 6.92
TSPM4	15.8 ± 0.20	19.5 ± 0.32	1183 ± 6.66
